# Pulmonary lymphangitic carcinomatosis and pathological fracture as the initial manifestations of occult gastric adenocarcinoma mimicking interstitial lung disease: a case report and review of the literature

**DOI:** 10.3389/fmed.2026.1932110

**Published:** 2026-09-23

**Authors:** Yan-Feng Yan, Wen-Xia Qi, Jie-Xiang Tian, Cai-Hong Wei, Yong-Hui Cui

**Affiliations:** 1 Department of Respiratory and Critical Care Medicine, The Fourth Affiliated Hospital of Gansu University of Chinese Medicine (Lanzhou Petrochemical General Hospital), Lanzhou, Gansu, China; 2 Department of Rheumatology and Bone Diseases, Affiliated Hospital of Gansu University of Chinese Medicine, Lanzhou, Gansu, China

**Keywords:** EBUS-TBNA, gastric adenocarcinoma, interstitial lung disease, pathological fracture, pulmonary lymphangitic carcinomatosis, signet-ring cell

## Abstract

**Background:**

Pulmonary lymphangitic carcinomatosis (PLC) clinically and radiologically mimics interstitial lung disease (ILD), posing diagnostic challenges when the primary malignancy is occult.

**Case presentation:**

A 33-year-old male presented with progressive cough, dyspnea, and a rib fracture following minimal trauma. Chest CT showed bilateral interlobular septal thickening and mediastinal lymphadenopathy. Endobronchial ultrasound-guided transbronchial needle aspiration (EBUS-TBNA) of subcarinal nodes confirmed metastatic adenocarcinoma, with immunohistochemistry (CK7+,TTF-1−, Napsin A−) suggesting gastrointestinal origin. Subsequent PET-CT and gastroscopy identified a 4.0 cm gastric angulus tumor, biopsied as moderately to poorly differentiated adenocarcinoma with signet-ring cells. Despite first-line and second-line chemotherapy with transient improvement, rapid progression ensued. The patient died on February 18, 2025, with an overall survival of approximately 6 months from metastatic diagnosis.

**Conclusion:**

This case illustrates that in young patients with ILD-like imaging and low-energy fractures, PLC warrants consideration even without gastrointestinal symptoms. Early use of EBUS-TBNA and PET-CT may facilitate timely diagnosis in appropriate clinical settings. Signet-ring cell-associated PLC carries a dismal prognosis, and transient responses should not be misconstrued as long-term benefit.

## 1 Introduction

Pulmonary lymphangitic carcinomatosis (PLC) is a distinctive pattern of intrapulmonary metastasis characterized by diffuse tumor cell infiltration along the pulmonary lymphatic vessels, accounting for approximately 6%–8% of all pulmonary metastases (1). Unlike the more common hematogenous metastases that present as discrete nodules, PLC typically manifests with non-specific respiratory symptoms (progressive dyspnea, dry cough) and diffuse reticulonodular or interstitial opacities on chest imaging—findings frequently indistinguishable from other forms of interstitial lung disease (ILD) (2, 3). The antemortem diagnosis of PLC remains exceedingly difficult (4), and the most common primary tumors giving rise to PLC are adenocarcinomas of the breast, lung, and stomach (5, 6). Among these, gastric adenocarcinoma presenting initially as PLC without overt gastrointestinal symptoms is rare, especially in young patients (7, 8). Here, we report a case of a 33-year-old male with occult gastric signet-ring cell adenocarcinoma whose initial presentation was refractory ILD complicated by a pathological rib fracture following minimal trauma, aiming to highlight diagnostic pitfalls and offer clinical lessons for similar challenging scenarios.

## 2 Case report

This case report was prepared in accordance with the CARE (Case Report) guidelines.

### 2.1 Patient information and clinical history

The patient was a 33-year-old male of Han ethnicity from Tianshui, Gansu Province, working as a chef. He had a 10-pack-year smoking history and was a chronic inactive hepatitis B carrier. He denied any gastrointestinal symptoms or weight loss.

**Initial Symptoms (June 8, 2024):** The patient developed paroxysmal severe cough without obvious precipitating factors, most prominent in the early morning and at night, with copious white sputum but no hemoptysis or fever. He received antitussive and mucolytic agents at a local clinic, with transient symptom relief.

**Critical Turning Point (June 14, 2024):** The patient experienced severe right chest pain after using a massager on his right chest wall, which he could not tolerate. Notably, the pressure applied was minimal and would not be expected to cause a fracture in healthy bone, which immediately raised the suspicion of a pathological fracture. The following day, he presented to our emergency department, denying any history of trauma such as falls or impacts. Emergency chest CT revealed “bilateral exudative changes, bilateral small pleural effusions, and a right 8th posterior rib fracture.” He was treated with moxifloxacin and methylprednisolone, with no improvement.

### 2.2 Admission examination and initial investigations

The patient was admitted to our department on July 17, 2024. Vital signs: temperature 36.3 °C, pulse 125 beats/min, respiratory rate 25 breaths/min, blood pressure 134/97 mmHg. At rest, his oxygen saturation (SpO₂) was 90% on room air, with central cyanosis. Lung auscultation revealed decreased breath sounds and scattered fine crackles in both lower lobes. Tenderness was elicited over the right lower chest wall, corresponding to the right 8th rib. Cardiac and abdominal examinations were unremarkable.

**Laboratory Findings:** White blood cell count 11.38 × 10⁹/L with eosinophilia (6%; reference 0.5%–5%), C-reactive protein 7.3 mg/L, procalcitonin 0.08 ng/mL, erythrocyte sedimentation rate 18 mm/h. Antinuclear antibody and interferon-gamma release assay for tuberculosis were negative. Serum tumor markers, including CEA, CA19-9, and CA72-4, were all within normal limits.

### 2.3 Diagnostic timeline

Table 1.

**Table 1:** Diagnostic and clinical timeline of the case.

Date	Event	Clinical Significance
June 8, 2024	Unexplained paroxysmal severe cough with white sputum.	Non-specific respiratory symptoms
June 14, 2024	Right chest pain after minimal pressure from a massager, intolerable.	Suspected pathological fracture
June 14, 2024	Emergency CT: bilateral exudates, small pleural effusions, right 8th posterior rib fracture. Treated with moxifloxacin + methylprednisolone, no response.	Imaging suggested pulmonary interstitial abnormality and pathological fracture
July 12, 2024	Cough and dyspnea recurred. Repeat CT unchanged. Eosinophilia 6%.	Anti-infective and anti-inflammatory treatment failure
July 17, 2024	Admitted to our department. SpO₂ 90% on room air, bilateral lower lobe crackles.	Impaired respiratory function
July 19, 2024	Bronchoscopy with BAL. BAL fluid t-NGS detected macrolide-resistant Mycoplasma pneumoniae. No malignant cells on cytology.	Diagnostic pitfall (confounding factor)
July 26, 2024	EBUS-TBNA (station 7 nodes): Metastatic adenocarcinoma. IHC: CK7(+), TTF-1(−), Napsin A(−).	Diagnostic breakthrough
August 8, 2024	PET-CT: Hypermetabolism in gastric antrum (SUVmax 12.3), both lungs (5.8), and multiple bone and lymph node metastases. Gastroscopy: 4.0 cm ulcerative mass at the gastric angulus; biopsy confirmed moderately to poorly differentiated adenocarcinoma (signet-ring cell).	Primary site and staging confirmed
August–September 2024	First-line chemotherapy: docetaxel + cisplatin + capecitabine, poor response.	First-line treatment failure
Sep 7, 2024	After 2 cycles of second-line chemotherapy (ramucirumab + nab-paclitaxel + tegafur), symptoms improved, SpO₂ rose to 92%, and imaging showed partial improvement.	Transient clinical improvement
October 2024 – January 2025	Progressive disease with worsening dyspnea, cachexia, and bone pain.	Disease progression
February 18, 2025	Patient died of multi-organ failure.	Overall survival ∼6 months

### 2.4 Key diagnostic findings

#### 2.4.1 Imaging

Initial Emergency CT (June 15, 2024): Predominantly showed bilateral exudative changes and small pleural effusions. In retrospect, subtle interlobular septal thickening may have been present, but the characteristic features of PLC were not yet definitive at this stage.

**HRCT (July 16–18, 2024):** Revealed diffuse bilateral irregular interlobular septal thickening, predominantly in the lower lobes, with a "crazy-paving" appearance in some areas and tree-in-bud opacities. Mediastinal lymphadenopathy (station 7, subcarinal) was noted. The characteristic HRCT findings of PLC became unequivocal on this examination. Serial CT changes are shown (Figure 1). Contrast-enhanced CT findings (Figure 2).

**Figure 1:**
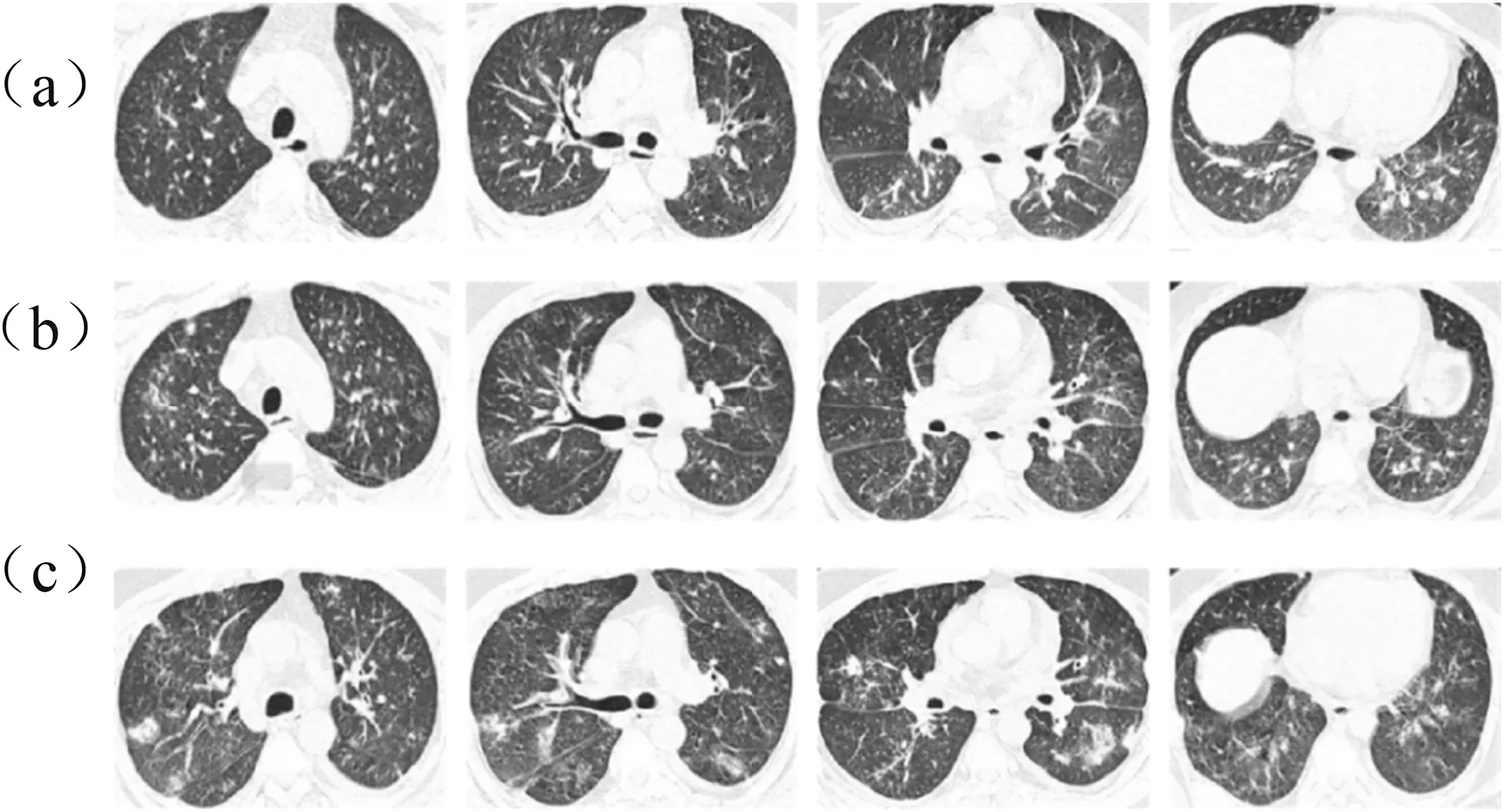
Serial chest CT images (axial view) of the same patient on **(a)** 15 June, **(b)**, 16 July and **(c)** 17 September.

**Figure 2:**
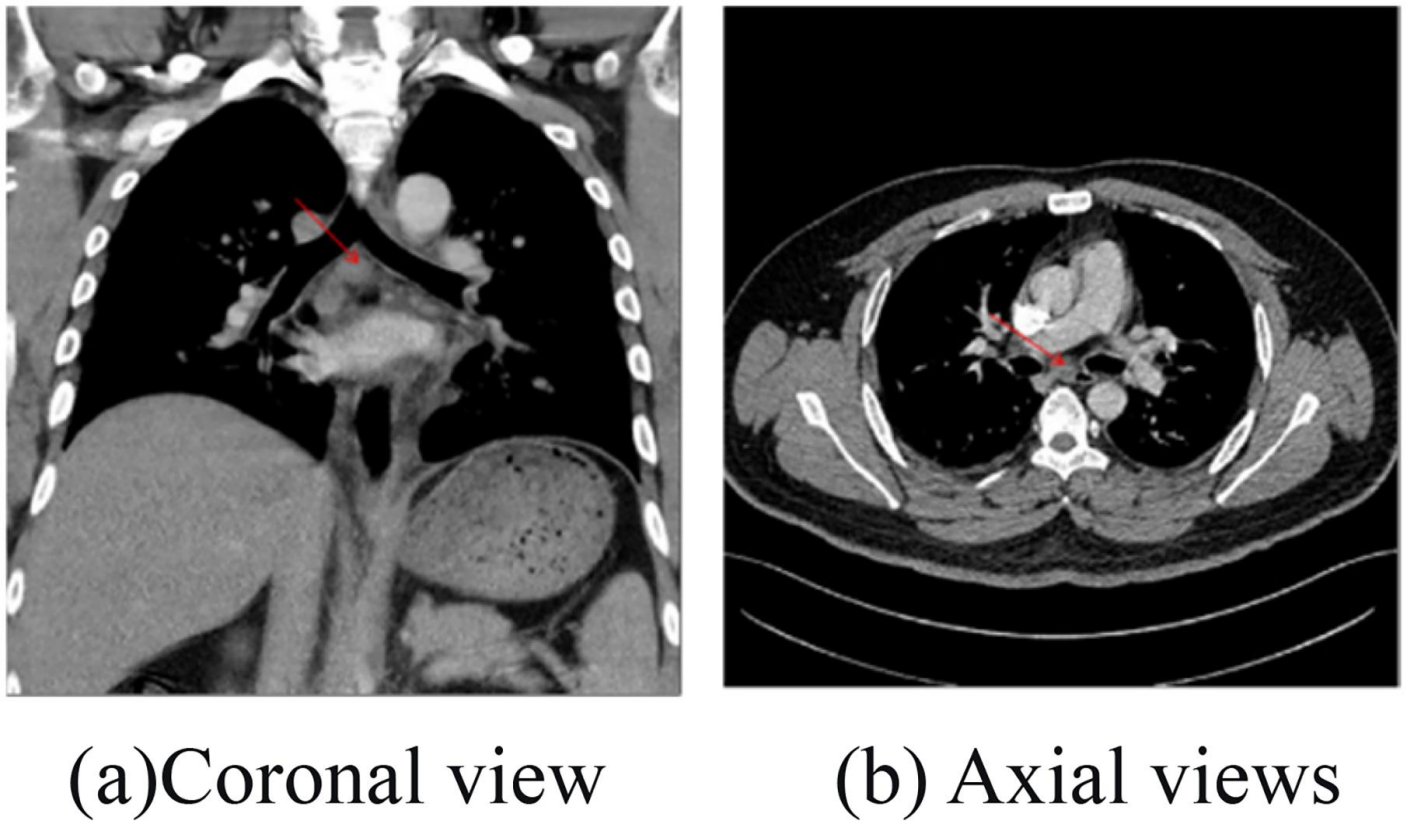
Contrast-enhanced chest CT image acquired on 18 July 2024.

Bronchoscopy: Bronchoscopy images (Figure 3).

**Figure 3:**
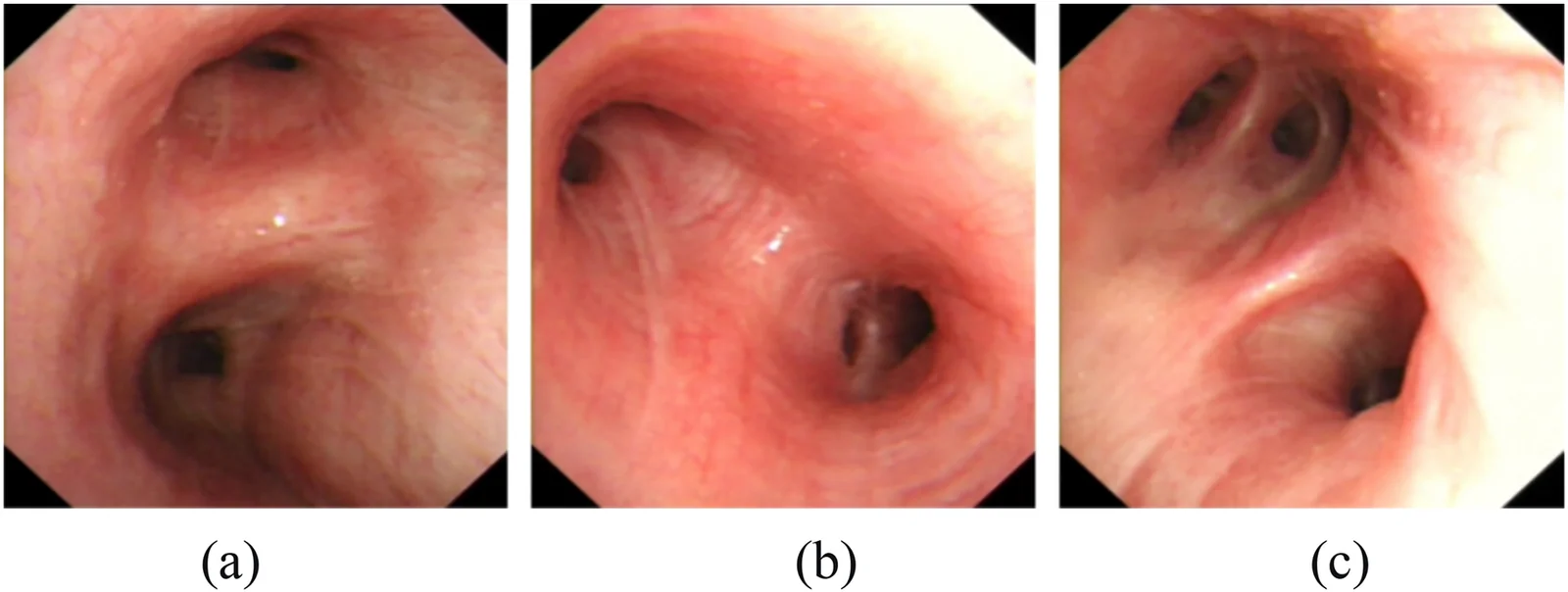
Bronchoscopic images. **(a)** Right upper lobe bronchus and intermediate bronchus. **(b)** Right middle and lower lobe bronchi (overview). **(c)** Right lower lobe bronchus (close-up).

**Pulmonary Function Tests:** Severe mixed ventilatory defect (FEV1/FVC = 58%, FVC%pred = 52%), with mildly reduced DLCO (62%pred).

#### 2.4.2 Pathology and immunohistochemistry

**EBUS-TBNA (July 26, 2024):** Histopathological examination of the station 7 lymph node biopsy revealed nests of atypical epithelial cells with an infiltrative growth pattern and prominent desmoplasia, consistent with metastatic adenocarcinoma. BAL fluid t-NGS detected macrolide-resistant M. pneumoniae (Figure 4). No malignant cells on cytology. EBUS-TBNA of station 7 nodes (Figure 5). Metastatic adenocarcinoma was confirmed (Figure 6). Immunohistochemically, the tumor cells were positive for CK7 and negative for TTF-1 and Napsin A, with a Ki-67 proliferation index of 30%. This immunophenotype (CK7+/TTF-1−/Napsin A−) effectively excluded primary lung adenocarcinoma and directed the diagnostic workup toward a gastrointestinal or pancreatobiliary origin, which was subsequently confirmed by PET-CT and gastroscopy. PET-CT revealed multi-site metastases (Figure 7).

**Figure 4:**
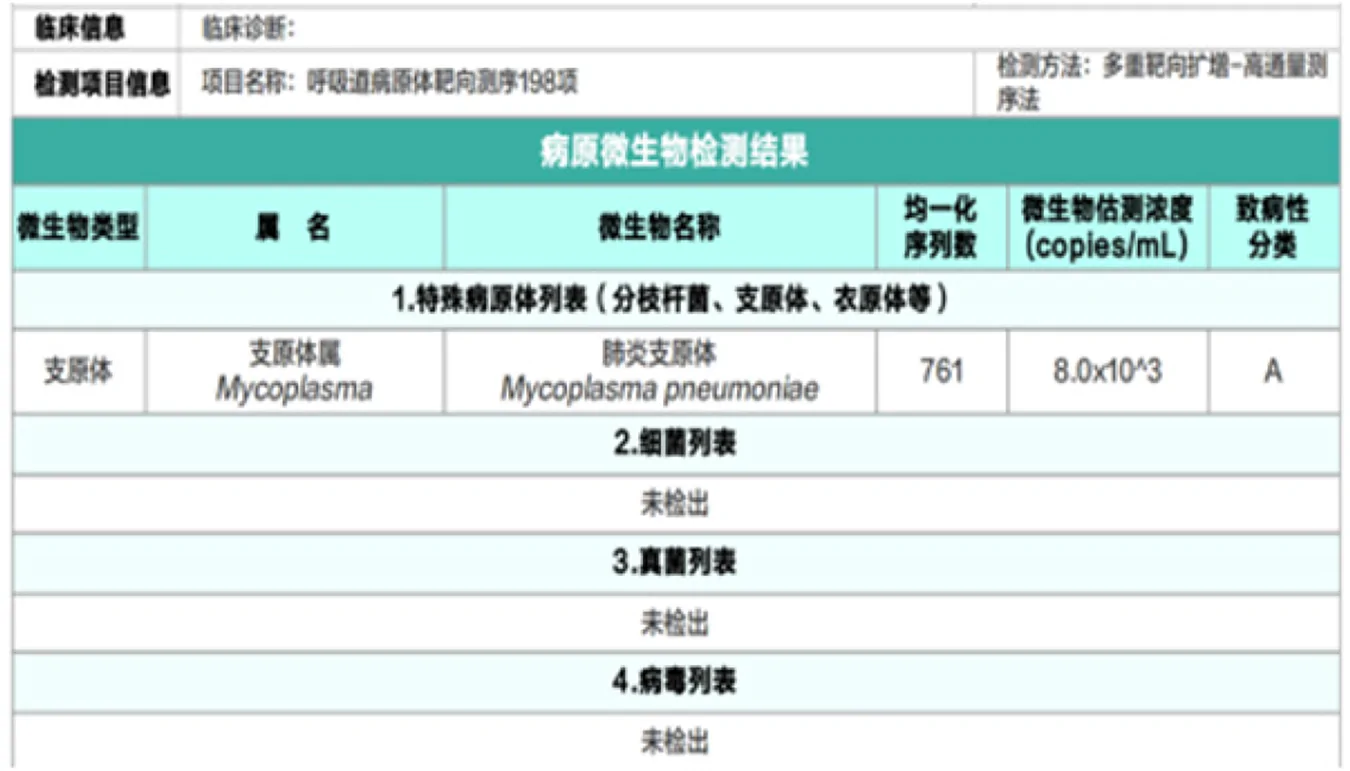
BAL fluid t-NGS: M. pneumoniae positive (sequence no. 761, 8.0 × 10³ copies/mL).

**Figure 5:**
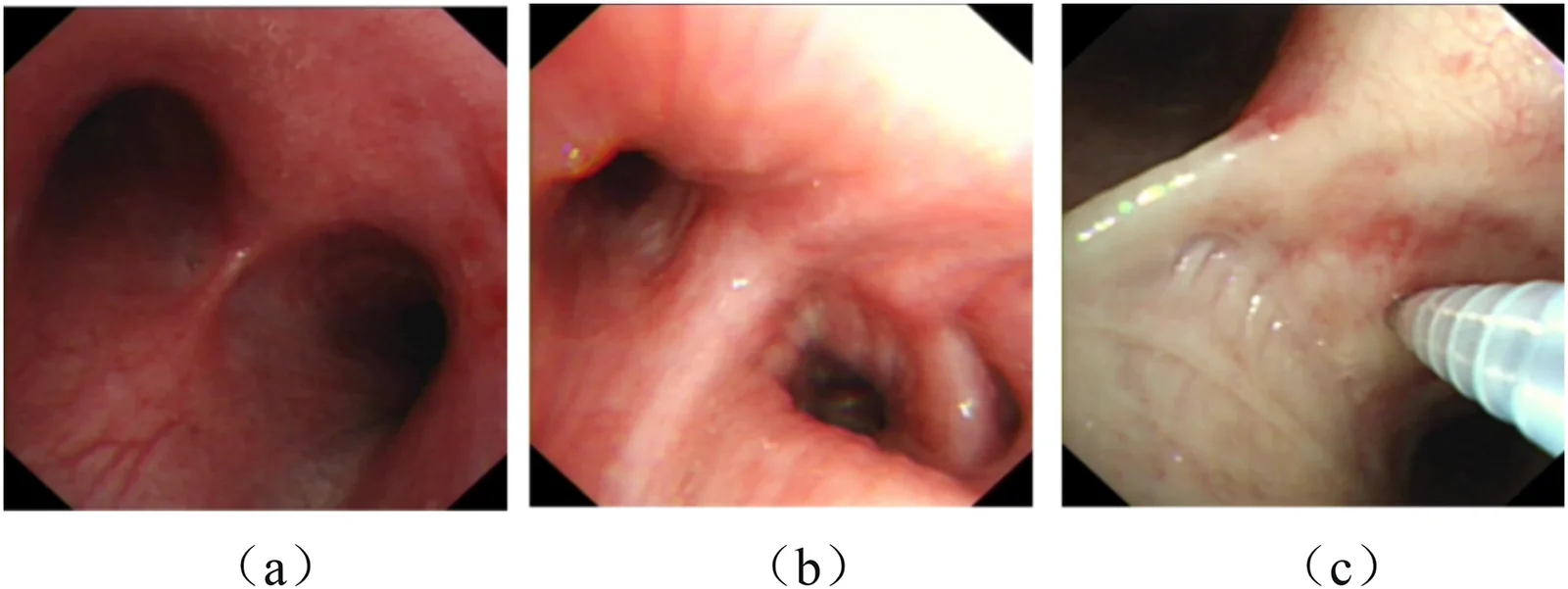
Bronchoscopic images and lymph node biopsy. **(a)** Carina. **(b)** Right middle and lower lobe bronchi. **(c)** Biopsy of the station 7 (subcarinal) lymph node.

**Figure 6:**
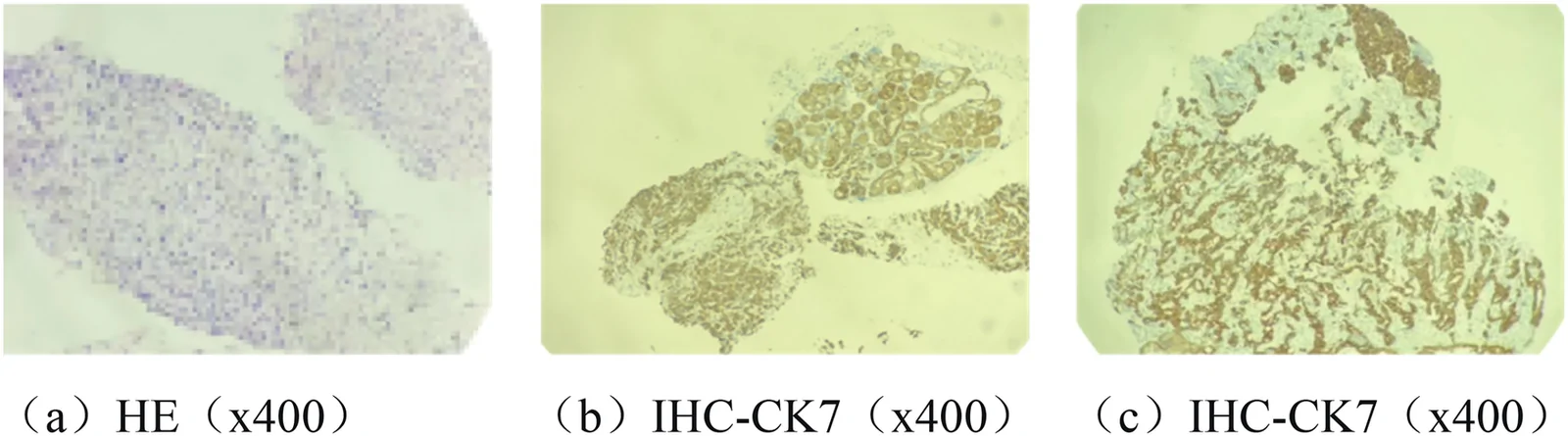
Histopathology and immunohistochemistry of the station 7 lymph node. **(a)** HE staining (×400). **(b,c)** Immunohistochemical staining for cytokeratin 7 (CK7).

**Figure 7:**
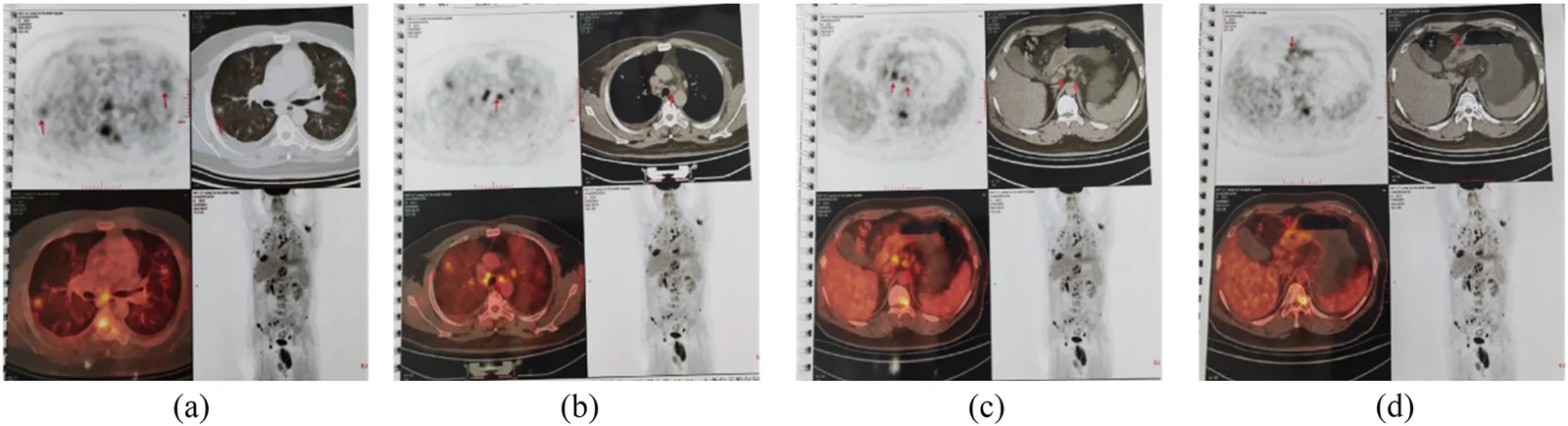
PET-CT. **(a)** Diffuse bilateral pulmonary opacities with increased FDG uptake, consistent with PLC involvement. **(b)** Multiple hypermetabolic lymph nodes in bilateral hilar, mediastinal, and supraclavicular areas. **(c)** Multiple hypermetabolic lymph nodes along the gastric lesser curvature, peripancreatic and retroperitoneal areas. **(d)** Gastric antral wall thickening with intense hypermetabolism and serosal penetration, corresponding to the primary tumor.

**Gastroscopic Biopsy (August 8, 2024):** A 4.0 cm ulcerative mass with irregular raised margins and a dirty base was found at the gastric angulus. Gastric angulus mass (Figure 8). Pathology confirmed a moderately to poorly differentiated adenocarcinoma with signet-ring cells. The histopathological and immunohistochemical (CK7) findings of the gastric mucosal tissue are shown in Figure 9. Immunohistochemistry: CK7 (+), CDX2 (-), HER2 (0), EBER (−).

**Figure 8:**
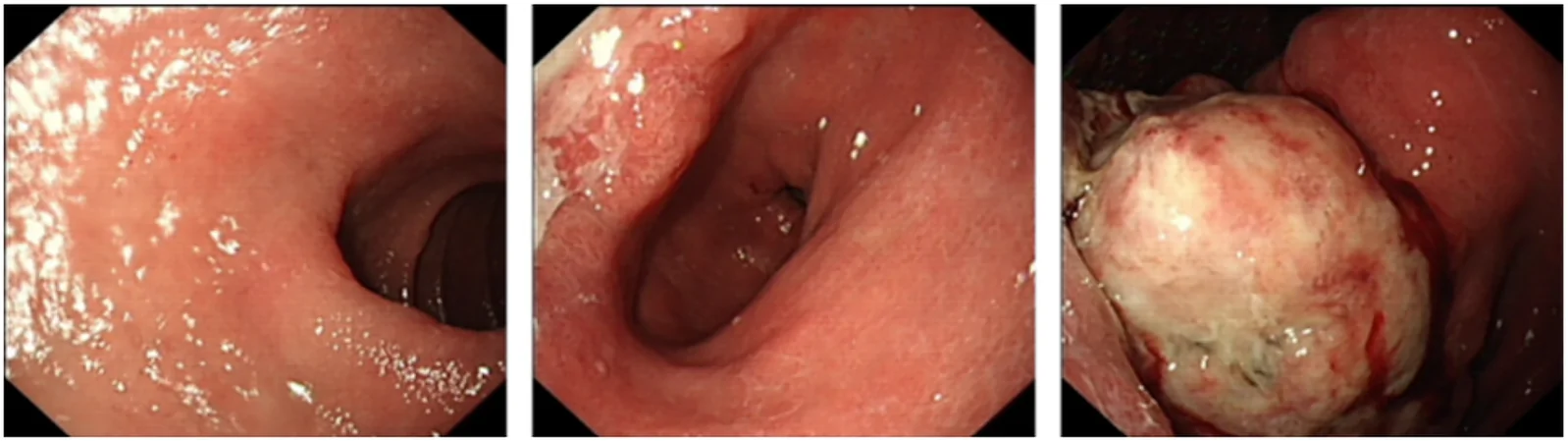
Gastroscopic view of the ulcerative lesion at the gastric angulus.

**Figure 9:**
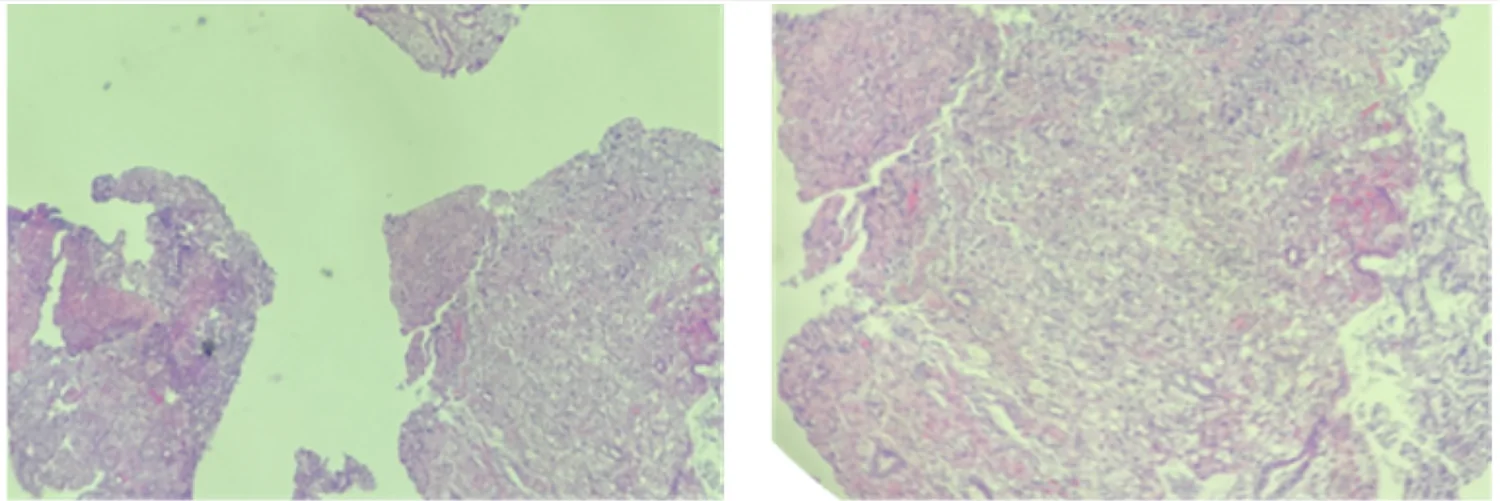
Histopathology of the gastric angulus biopsy (HE ×400).

**Final Diagnosis:** Gastric adenocarcinoma, cT4aN3bM1, stage IVB (AJCC 8th edition); secondary pulmonary lymphangitic carcinomatosis; multiple bone metastases with pathological right 8th posterior rib fracture; ECOG PS 1.

### 2.5 Treatment and follow-up

Following the diagnosis of stage IVB gastric adenocarcinoma with PLC and bone metastases, the patient received first-line palliative chemotherapy with docetaxel (75 mg/m², day 1), cisplatin (75 mg/m², day 1), and capecitabine (1,000 mg/m² twice daily, days 1–14) on a 21-day cycle. This regimen was chosen based on its established efficacy in HER2-negative advanced gastric cancer and the patient's preserved ECOG PS 1. After two cycles, restaging CT showed stable disease (SD) per RECIST 1.1, with no significant tumor reduction. Given the lack of HER2 overexpression and declining tolerance to cisplatin, treatment was switched to ramucirumab (8 mg/kg on days 1 and 15) plus nab-paclitaxel (100 mg/m² on days 1, 8, and 15) and tegafur on a 28-day cycle, a standard second-line option for advanced gastric cancer.

After two cycles of second-line therapy (September 7, 2024), the patient showed transient clinical improvement: cough and dyspnea decreased, SpO₂ rose from 90% to 92%, and chest CT demonstrated partial resolution of interstitial changes. Per RECIST 1.1, this was classified as stable disease with symptomatic improvement rather than partial response. No grade ≥3 toxicity occurred; only grade 1 fatigue and nausea were noted. The patient reported improved dyspnea-related quality of life, with less exertional limitation and better sleep.

However, from October 2024 onward, the patient experienced progressive dyspnea, cachexia, and bone pain. Chest CT in January 2025 showed progressive disease (PD) with worsening PLC infiltrates and new osteolytic lesions. Due to deteriorating performance status (ECOG PS 3–4), no further chemotherapy was administered. The patient received best supportive care and died of multi-organ failure on February 18, 2025. Overall survival from diagnosis was approximately 6 months.

### 2.6 Patient perspective

The patient's parents provided the following account. “He was only 33 and had always been healthy. When he broke a rib just from using a massager, we knew something was terribly wrong. The weeks of tests and uncertainty were agonizing. When bronchoscopy detected Mycoplasma pneumoniae, we felt a brief relief—finally an answer—but that hope faded when treatment failed and his breathing worsened. Hearing the word “cancer” with no prior stomach symptoms was devastating. During the short period of chemotherapy improvement, we allowed ourselves to hope again, though we knew it might not last. When the doctors told us further treatment would no longer help, their honesty and compassion helped us prepare for what was coming. We share our story so that other young people and their families may not have to go through such a long and confusing journey to diagnosis.”

## 3 Discussion

This case describes a highly instructive presentation of occult gastric signet-ring cell adenocarcinoma in a 33-year-old male, manifesting solely as PLC with an unexplained pathological rib fracture. What makes this case particularly instructive is not the PLC manifestation itself, but rather the convergence of multiple diagnostic challenges in a single patient. The diagnostic journey of this patient offers several important clinical lessons.

### 3.1 Literature search strategy

To contextualize our findings, we performed a targeted literature review. We searched PubMed and CNKI databases from inception to January 2026 using the following keywords and their combinations: “pulmonary lymphangitic carcinomatosis,” “gastric adenocarcinoma,” “signet-ring cell,” “pathological fracture,” and “interstitial lung disease.” We included case reports, case series, and original articles reporting gastric cancer presenting initially with PLC or pathological fractures. References of retrieved articles were also screened for additional relevant publications. Given the rarity of this entity, a systematic quantitative synthesis was not feasible; instead, we summarized the key clinical features and diagnostic approaches from the available literature to support the discussion of our case.

### 3.2 From imaging to diagnosis: a stepwise approach

#### 3.2.1 Imaging characteristics and differential diagnosis

The HRCT findings in this case demonstrated nodular ILST, predominantly in the lower lobes, appearing as beaded or irregular septal thickening—one of the most characteristic imaging features of PLC (9). Beyond PLC, other conditions such as sarcoidosis, silicosis, and diffuse alveolar septal amyloidosis may also present with nodular ILST, though each has distinct distribution and associated findings. Sarcoidosis typically shows perilymphatic distribution predominantly in the mid-to-upper lung zones, often with bilateral hilar lymphadenopathy, while silicosis predominantly affects the posterior upper lobes with eggshell calcification of hilar nodes. Pasławski et al. noted that lung architecture remains preserved in PLC, whereas architectural distortion is common in sarcoidosis—a feature that aids in their differentiation (9).

Several other conditions can present with bilateral nodular ILST and should be considered in the differential diagnosis. Cryptogenic organizing pneumonia (COP) typically manifests with bilateral patchy consolidations or ground-glass opacities, often with a subpleural or peribronchial distribution. Although COP may show associated interstitial thickening, it usually responds dramatically to corticosteroids—a feature absent in our patient, who failed to improve with methylprednisolone. Lymphoma or lymphoproliferative disorders can produce pulmonary infiltrates with mediastinal lymphadenopathy. However, lymphomatous involvement typically presents with bulkier nodal masses, and the micronodular/interlobular septal thickening pattern is less prominent than in PLC. Moreover, the immunohistochemical profile (CK7+,TTF-1−,Napsin A−) would be unusual for lymphoma, which is typically negative for cytokeratins.

Associated imaging findings are critical for differential diagnosis in the context of nodular ILST. PLC is frequently accompanied by bronchovascular bundle thickening, centrilobular micronodules, mediastinal/hilar lymphadenopathy, and pleural effusion (10, 11). In this case, the presence of nodular ILST with bronchovascular bundle thickening, mediastinal (station 7) lymphadenopathy, and a right 8th posterior rib fracture—a triad of nodular ILST, mediastinal lymphadenopathy, and bone metastasis—strongly suggested PLC. Jreige et al. demonstrated that PET-CT quantitative parameters (e.g., peri-tumoral SUVmax≥2.1) achieve superior diagnostic performance for PLC (sensitivity 86%–97%, specificity 84%–100%) compared to HRCT morphology alone (12). In this case, PET-CT ultimately revealed multi-organ metastases, thereby linking the pulmonary, gastrointestinal, and skeletal findings. Therefore, the diagnostic reasoning proceeded through systematic exclusion of these alternatives, with the combination of HRCT findings, immunohistochemistry, and PET-CT collectively pointing toward PLC of gastric origin.

#### 3.2.2 Immunohistochemical profiling

In terms of diagnostic strategy, EBUS-TBNA was pivotal in this case. Aspiration of the subcarinal lymph nodes not only provided histologic confirmation of metastatic adenocarcinoma in a minimally invasive manner but also, through immunohistochemistry, directed the diagnostic workup toward a gastrointestinal origin, guiding subsequent PET-CT and gastroscopy (13). The sensitivity of EBUS-TBNA for detecting mediastinal lymph node metastases has been reported to be as high as 91.2%, with a specificity of 100% (14). In patients with ILD of unknown etiology who have accessible mediastinal or hilar lymphadenopathy, EBUS-TBNA offers a valuable minimally invasive diagnostic option that may expedite pathological confirmation. However, we acknowledge that this approach is contingent upon the presence of targetable nodal enlargement; in its absence, alternative biopsy routes—such as CT-guided percutaneous lung biopsy or PET-CT-directed biopsy of hypermetabolic lesions—should be considered.

The immunophenotype CK7+/TTF-1−/Napsin A−from the EBUS-TBNA specimen was crucial in excluding primary lung adenocarcinoma (15, 16), as TTF-1 and Napsin A are highly sensitive and specific markers for pulmonary origin (TTF-1: sensitivity∼70%–80%, specificity∼90% for lung adenocarcinoma; Napsin A: sensitivity∼80%, specificity∼85%) (17, 18). However, it is important to acknowledge that this profile is not pathognomonic for gastric cancer. CK7 positivity with TTF-1 and Napsin A negativity can be seen in adenocarcinomas from a variety of primary sites, including breast, pancreas, biliary tract, ovary, and upper gastrointestinal tract (18). Therefore, this immunophenotype serves as a directional “traffic light”—effectively ruling out lung origin while broadly pointing toward a gastrointestinal or pancreatobiliary primary—but does not provide a definitive tissue-of-origin diagnosis.

Ideally, additional immunohistochemical markers would have helped narrow the differential further. For gastric adenocarcinoma, CDX2 (a homeobox transcription factor expressed in intestinal epithelium) is positive in approximately 70%–90% of gastric cancers, particularly the intestinal subtype, though it may also be expressed in colorectal, pancreatic, and biliary adenocarcinomas (19). CK20 is less consistently expressed in gastric cancer (40%–60%) and is more characteristic of colorectal origin (20). More recently, Claudin 18.2 has emerged as a highly sensitive marker for gastric adenocarcinoma, with expression reported in over 80% of cases, and has gained clinical relevance as a therapeutic target (21, 22). SATB2 is useful for distinguishing colorectal from upper gastrointestinal origin, as it is typically negative in gastric cancer (23). Unfortunately, the limited cellularity of the EBUS-TBNA specimen and the small biopsy size from the gastric lesion precluded comprehensive marker profiling in this case. This represents a limitation of our diagnostic workup. Nevertheless, the CK7+/TTF-1−/Napsin A− result, combined with the clinical context (young patient with ILD-like imaging, pathological fracture, and mediastinal lymphadenopathy), was sufficiently compelling to guide PET-CT and gastroscopy, which ultimately confirmed the gastric primary. In future cases, judicious use of additional markers such as CDX2, CK20, and Claudin 18.2 on available biopsy material is encouraged to enhance diagnostic precision, particularly when the primary site remains unclear despite imaging.

#### 3.2.3 Clinical implications of molecular profiling

Beyond its diagnostic utility, comprehensive molecular profiling has become increasingly critical for guiding treatment decisions in advanced gastric adenocarcinoma. CLDN18.2, a tight junction protein normally restricted to gastric mucosal epithelium, is overexpressed in a substantial proportion of gastric cancers. Notably, CLDN18.2 positivity is particularly prevalent in signet-ring cell carcinoma (SRCC). One study reported CLDN18.2 positivity in 49.2% of SRCC cases overall and 50.9% in those with lymphatic metastasis (24); another series found CLDN18.2 positivity in 62% of SRCC cases, significantly higher than in non-SRCC (39%, *p* < 0.001) (25).

This high prevalence has direct therapeutic implications. The phase III SPOTLIGHT and GLOW trials established zolbetuximab—a CLDN18.2-directed cytolytic antibody—combined with chemotherapy as a new standard first-line treatment for HER2-negative, CLDN18.2-positive advanced gastric or gastroesophageal junction adenocarcinoma (26). In SPOTLIGHT (zolbetuximab + mFOLFOX6), median progression-free survival (PFS) was 10.6 months vs. 8.7 months with placebo (HR 0.751; 95% CI: 0.598–0.942), and median overall survival (OS) was 18.2 months vs. 15.5 months (HR 0.750; 95% CI: 0.601–0.936) (26). In GLOW (zolbetuximab + CAPOX), median PFS was 8.2 months vs. 6.8 months (HR 0.687; 95% CI: 0.544–0.866), and median OS was 14.4 months vs. 12.2 months (HR 0.771; 95% CI: 0.615–0.965) (27). Based on these results, the FDA approved zolbetuximab (Vyloy) in October 2024 for first-line use in CLDN18.2-positive, HER2-negative advanced gastric cancer.

Additionally, PD-L1 testing with CPS (combined positive score) is now standard for identifying patients eligible for immune checkpoint inhibitors in the first-line setting, particularly in combination with chemotherapy. However, in our case, the limited biopsy material precluded both CLDN18.2 and PD-L1 testing—a missed opportunity that, with today's standard of care, would have potentially offered additional therapeutic options. This underscores the importance of obtaining adequate tissue at initial diagnosis to enable comprehensive molecular characterization, which is essential for personalized treatment planning in advanced gastric cancer.

### 3.3 Diagnostic pitfalls: from fracture to misleading infection

The most critical early clue in this case was a rib fracture occurring after minimal trauma. The patient sustained a fracture from merely using a massager—pressure that would not normally be expected to cause a fracture in healthy bone—a finding that strongly suggests a pathological fracture. Bone metastasis is a common complication of advanced gastric cancer, with approximately 10%–15% of cancer patients presenting with pathological fracture as their initial symptom (28). Although gastric cancer presenting with bone metastasis as the first manifestation is relatively uncommon, it is characterized by an occult primary site and prolonged diagnostic delay. Bone metastasis as the initial presentation of gastric cancer is considered extremely rare (29–31). These cases may first manifest as unexplained pathological fractures, persistent bone pain, or diffuse osteolytic lesions, typically without gastrointestinal symptoms. For young patients, a fracture occurring with minimal force should first raise suspicion of a pathological fracture and prompt systematic evaluation for an underlying malignancy.

Another diagnostic challenge was the incidental detection of macrolide-resistant Mycoplasma pneumoniae by BAL fluid t-NGS. This finding could easily lead clinicians to anchor on a diagnosis of “refractory mycoplasma pneumonia.” However, several lines of evidence suggest that M. pneumoniae was a bystander rather than the causative pathogen. First, the patient lacked typical clinical features of M. pneumoniae pneumonia, such as high fever or significant systemic symptoms; his inflammatory markers (procalcitonin 0.08 ng/mL, CRP 7.3 mg/L) remained relatively low throughout the course, which is atypical for a clinically significant infection. Second, eosinophilia (6%) and bilateral interstitial opacities are more consistent with a non-infectious etiology than with M. pneumoniae pneumonia. Third, and most importantly, the disease responded only transiently to systemic chemotherapy and progressed when treatment was paused—a pattern incompatible with an infectious process. Therefore, while M. pneumoniae DNA was present in the BAL fluid, its clinical significance in this case was most likely as an incidental colonization rather than a contributory infection.

These two diagnostic pitfalls—a fracture that was initially overlooked and a positive molecular test that was initially overinterpreted—collectively delayed the correct diagnosis. This case underscores an important principle: clinical findings must be interpreted within the broader context, and molecular test results, while valuable, should not override clinical judgment when the treatment response is inconsistent with the detected pathogen.

### 3.4 The triple diagnostic challenge

This case presented a triple diagnostic challenge that collectively delayed the correct diagnosis: (1)Age factor—the patient was only 33 years old, and clinicians were reluctant to consider gastric cancer due to his youth, contributing substantially to the diagnostic delay; (2)Atypical symptoms—the complete absence of gastrointestinal manifestations (nausea, abdominal pain, melena) directed diagnostic efforts toward the respiratory system; (3)Radiological confounding—the ILD-mimicking pattern and incidental detection of Mycoplasma pneumoniae further masked the underlying malignancy.

In recent years, the incidence of gastric cancer in young adults (≤40 years) has been rising (32). Such tumors are characterized by poor differentiation (higher proportion of signet-ring cell histology), aggressive behavior, early metastasis (33), and atypical clinical presentations, leading to high rates of misdiagnosis (34).

Several similar cases have been reported in the literature. Moubax et al. described a 29-year-old female presenting with progressive dyspnea who was ultimately diagnosed with gastric signet-ring cell carcinoma with PLC (7). Other reports have described young females with gastric-origin PLC presenting with progressive dyspnea, who were not suspected of having gastric cancer due to their young age (8). However, in those cases, the diagnostic process was not confounded by an additional misleading factor—namely, a positive molecular test for an infectious pathogen—which in our case substantially delayed the correct diagnosis. This distinction highlights that the diagnostic pitfalls in PLC extend beyond radiological mimicry and may be compounded by incidental laboratory findings. Beyond these diagnostic challenges, the pathological features of SRCC itself also contribute to its unique clinical behavior. Signet-ring cell carcinoma (SRCC) is characterized by low cohesiveness and abundant mucin production (35). This low cohesiveness, resulting from disrupted cell–cell adhesion, forms the pathological basis for its high propensity for lymphatic invasion and metastasis. Furthermore, the overexpression of lymphangiogenic factors such as vascular endothelial growth factor-C (VEGF-C) may facilitate tumor spread along lymphatic channels (36). These features collectively contribute to the recognized association between SRCC and pulmonary lymphangitic carcinomatosis (PLC) (37).

The complete absence of gastrointestinal symptoms in this patient underscores that the absence of digestive complaints does not exclude advanced gastric cancer. This case serves as a reminder that age alone should not exclude malignancy from the differential diagnosis. For young patients presenting with multi-system involvement (lung, bone, lymph nodes) and refractory ILD, occult gastric cancer deserves consideration even in the absence of gastrointestinal symptoms.

### 3.5 Prognosis of pulmonary lymphangitic carcinomatosis

PLC carries a dismal prognosis, with reported median survival of approximately 2–7 months. Consistent with this, our patient survived only approximately 6 months from diagnosis. Notably, he experienced transient clinical improvement with symptomatic relief and improved oxygenation (SpO₂ from 90% to 92%) during second-line chemotherapy with ramucirumab and nab-paclitaxel. However, this improvement did not translate into long-term survival benefit.

The limited and short-lived response to ramucirumab in this case warrants further biological explanation. In the phase III RAINBOW trial, which established ramucirumab plus paclitaxel as a standard second-line regimen for advanced gastric cancer, the addition of ramucirumab showed less benefit in diffuse-type gastric cancer (DGC) than in intestinal-type gastric cancer (IGC), both in terms of progression-free survival (PFS; HR 0.70, 95% CI 0.52–0.93 for DGC vs. HR 0.53, 95% CI 0.41–0.69 for IGC) and overall survival (OS; HR 0.86, 95% CI 0.64–1.15 for DGC vs. HR 0.71, 95% CI 0.53–0.93 for IGC) (38). Real-world data have corroborated these findings: in a retrospective cohort of 179 patients receiving second-line treatment, paclitaxel/ramucirumab was independently associated with OS benefit only in patients with intestinal-type histology (aHR 0.44, 95% CI 0.22–0.88), whereas no significant OS benefit was observed in diffuse-type histology (aHR 1.71, 95% CI 0.79–3.68) (39). Preclinically, the dependency on angiogenesis is likely to be higher in IGC than in DGC (21). Signet-ring cell carcinoma—characterized by low cohesiveness, abundant mucin production, and a propensity for lymphatic rather than hematogenous spread—may render tumors less dependent on VEGF-driven angiogenesis, thereby diminishing the efficacy of anti-VEGFR2 agents such as ramucirumab. In our patient, the presence of extensive pulmonary lymphangitic spread and diffuse bone metastases, coupled with signet-ring cell pathology, likely contributed to the limited and short-lived clinical benefit from second-line therapy.

For patients with PLC, short-term symptomatic improvement should not be misinterpreted as a signal of long-term benefit; treatment goals should focus on symptom palliation and maintenance of quality of life, and prognostic communication should be initiated early with patients and their families.

### 3.6 Limitations and clinical takeaways

As a single case report, our findings have limited generalizability and cannot establish causality. The diagnostic framework we propose requires prospective validation in larger cohorts before its predictive value can be assessed. Publication bias should also be considered, as instructive cases are more likely to be reported, potentially overestimating the utility of certain diagnostic strategies. Therefore, our observations should be interpreted as hypothesis-generating rather than practice-changing.

This report has several limitations. As a single case observation, it cannot establish causality. The diagnosis relied on EBUS-TBNA of mediastinal lymph nodes and gastroscopy; no lung parenchymal biopsy was performed to obtain direct histopathological evidence of lymphangitic tumor spread within the lung interstitium. In retrospect, transbronchial lung cryobiopsy (TBLC) could have been considered as an alternative approach. TBLC has emerged as a superior minimally invasive technique to conventional forceps biopsy for interstitial lung diseases, yielding significantly larger tissue specimens (often >5 mm in diameter) without crush artifacts, thereby preserving alveolar architecture and enabling more accurate histopathological interpretation (40–42). A meta-analysis of 68 studies involving over 6,300 patients reported an overall diagnostic yield of TBLC of 82.3% (95% CI: 78.9%–85.2%) for achieving a definite or high-confidence diagnosis based on multidisciplinary discussion, with a histopathological diagnostic yield of 72.5% (95% CI: 67.7%–76.9%) (43). A systematic review and meta-analysis further demonstrated that the pooled multidisciplinary diagnostic yield of TBLC (84.3%, 95% CI 81.7%–86.5%) is significantly higher than that of conventional transbronchial forceps biopsy (35.3%, 95% CI 21.3%–52.5%) and approaches that of surgical lung biopsy (88.6%, 95% CI 83.4%–92.3%) (44). For PLC specifically, TBLC has been shown to clearly demonstrate tumor cell infiltration along lymphatic vessels, a finding that may be missed on conventional forceps biopsy or bronchoalveolar lavage (45). Furthermore, TBLC specimens are suitable for multiplex molecular testing, potentially enabling simultaneous histologic diagnosis and biomarker analysis. However, TBLC is not without limitations: it requires general anesthesia, operator expertise, and carries risks of pneumothorax (approximately 5%) and moderate-to-severe bleeding (approximately 12%) (46). In our case, the presence of accessible mediastinal lymphadenopathy made EBUS-TBNA a more direct and less invasive route to diagnosis. Nevertheless, for future patients with suspected PLC but without targetable lymphadenopathy, TBLC should be considered as a valuable minimally invasive alternative to surgical lung biopsy for obtaining definitive histopathological confirmation.

Comprehensive molecular profiling—including CLDN18.2, PD-L1, and MSI status—was not performed due to limited biopsy material, precluding assessment of targeted therapy (e.g zolbetuximab for CLDN18.2-positive patients) or immunotherapy opportunities. This represents a missed therapeutic opportunity under current standard of care and underscores the critical importance of obtaining adequate tissue at initial diagnosis for comprehensive biomarker testing.

Despite these limitations, several clinical takeaways emerge from this case. PLC should be considered in patients with diffuse interlobular septal thickening mimicking ILD, especially when accompanied by low-energy fractures. The combination of EBUS-TBNA and PET-CT can facilitate timely diagnosis when mediastinal lymphadenopathy is present. Age alone should not exclude gastric cancer from the differential diagnosis in young patients with multi-system involvement and refractory ILD-like imaging. Finally, even transient therapeutic responses may not translate into long-term survival benefit in signet-ring cell-associated PLC, underscoring the importance of early prognostic communication with patients and families.

## Data Availability

The original contributions presented in the study are included in the article/Supplementary Material, further inquiries can be directed to the corresponding author.
